# Supplementary material for: Disruption of Irisin Dimerization by FDA-Approved Drugs: A Computational Repurposing Approach for the Potential Treatment of Lipodystrophy Syndromes
Source: Int J Mol Sci. 2023 Apr 20;24(8):7578. doi: 10.3390/ijms24087578 (PMC10145865; doi:10.3390/ijms24087578)
Supplement: Supplementary file 1 [file ijms-24-07578-s001.zip › ijms-2329030-supplementary.pdf]

International Journal of
Molecular Sciences

MDPI

Supplementary material

Disruption of Irisin Dimerization by FDA-approved Drugs. A Computational Repurposing Approach for the Potential Treatment of Lipodystrophy Syndromes

Lorenzo Flori [1,a], Simone Brogi [1,2,a,*], Hajar Sirous[2] and Vincenzo Calderone[1]

[1] Department of Pharmacy, University of Pisa, Via Bonanno 6, 56126 Pisa, Italy
[2] Bioinformatics Research Center, School of Pharmacy and Pharmaceutical Sciences, Isfahan University of Medical Sciences, 81746-73461 Isfahan, Iran
[a] These authors contributed equally to this work
* Correspondence: simone.brogi@unipi.it

Table of Contents

Figure S1 page S2

Irisin/Diquafosal

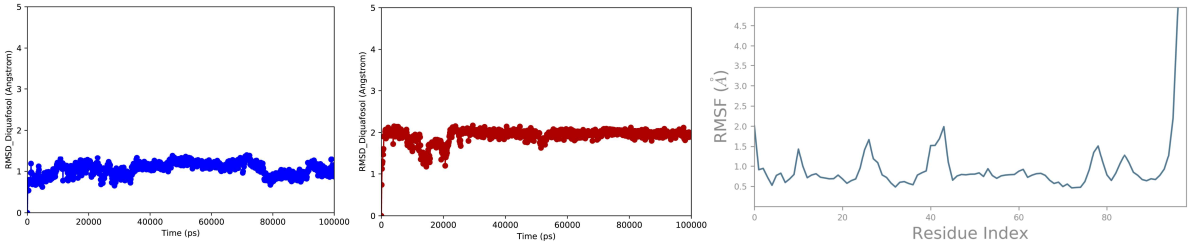

Irisin/Isepamicin

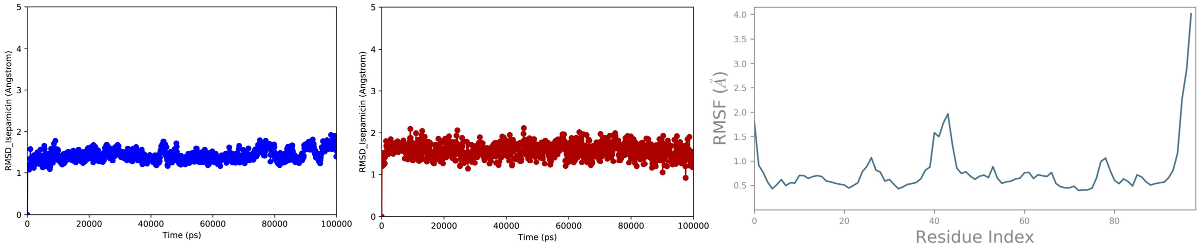

Irisin/Iohexol

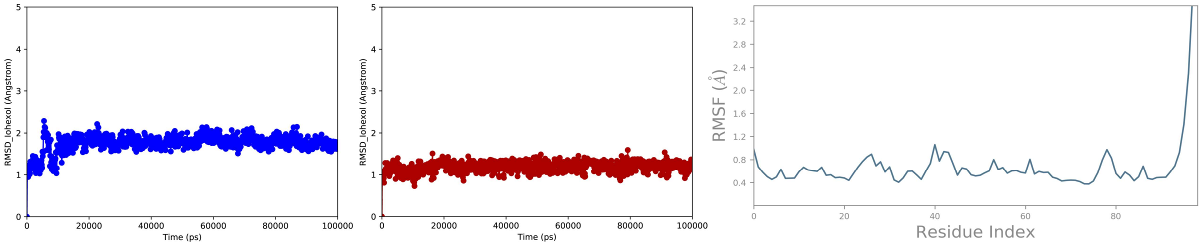

Irisin/Voglibose

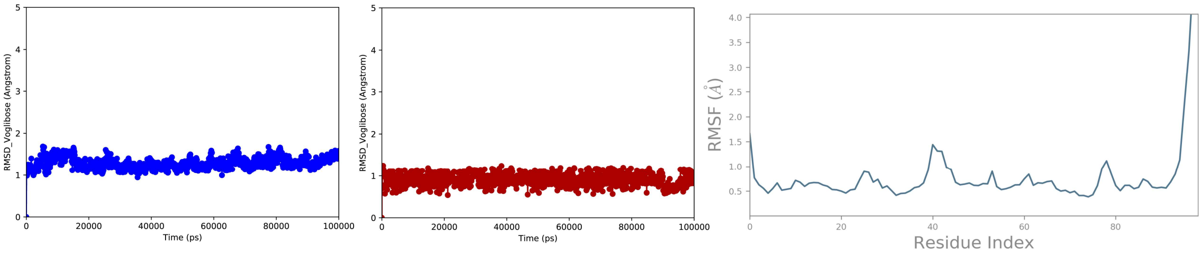

Irisin/Troxerutin

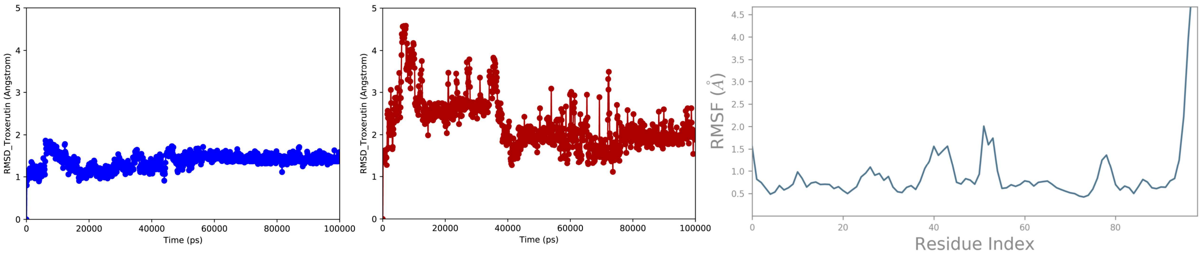

Irisin/Paromomycin

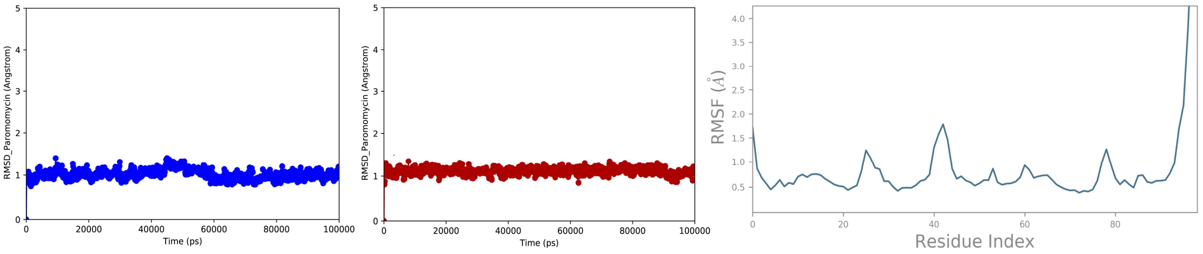

Continue

Irisin/Amikacin

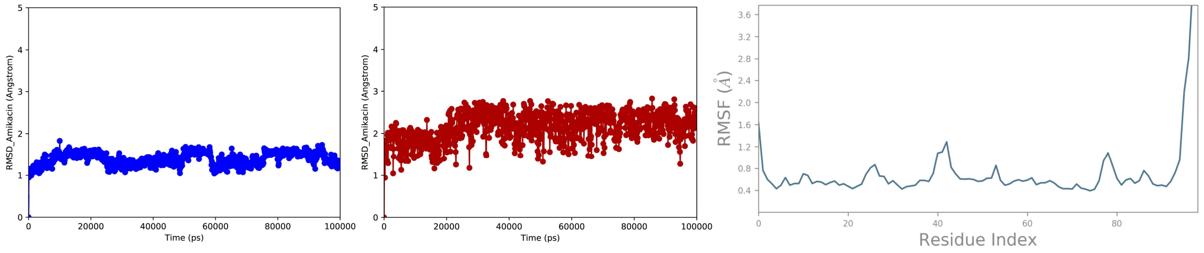

Irisin/Benserazide

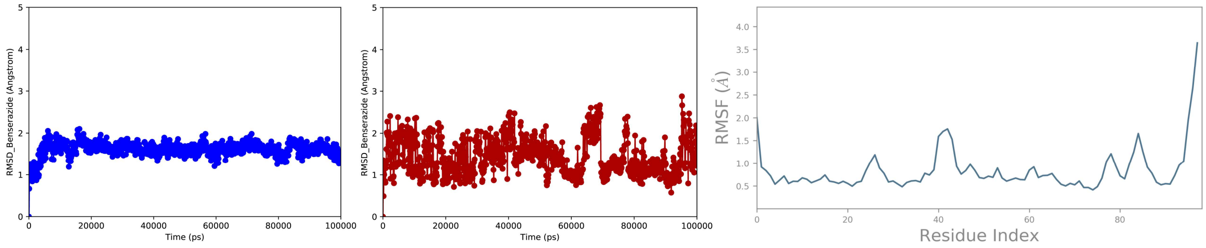

Irisin/Lactulose

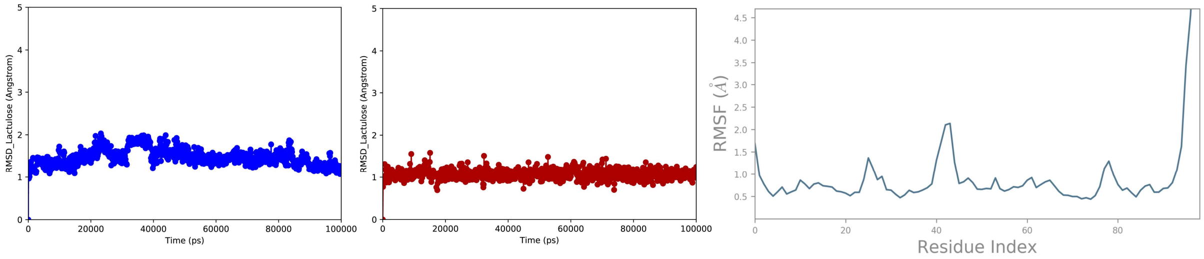

Irisin/Acarbose

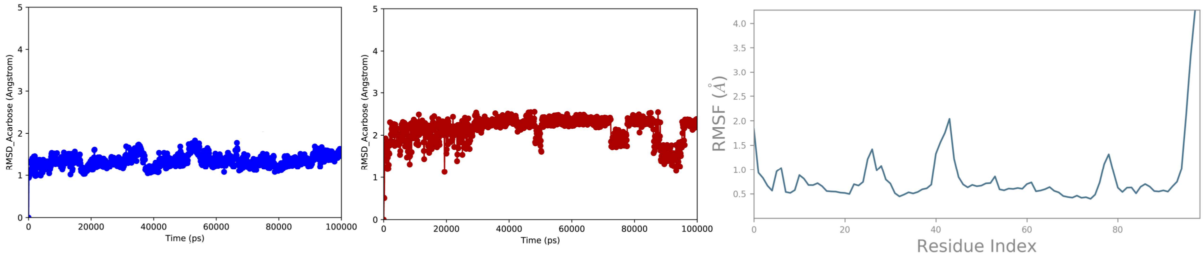

Irisin/Hesperidin

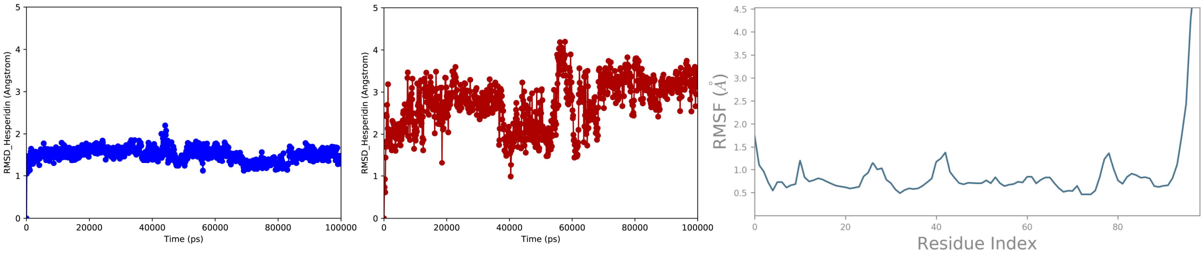

Irisin/Astragaloside IV

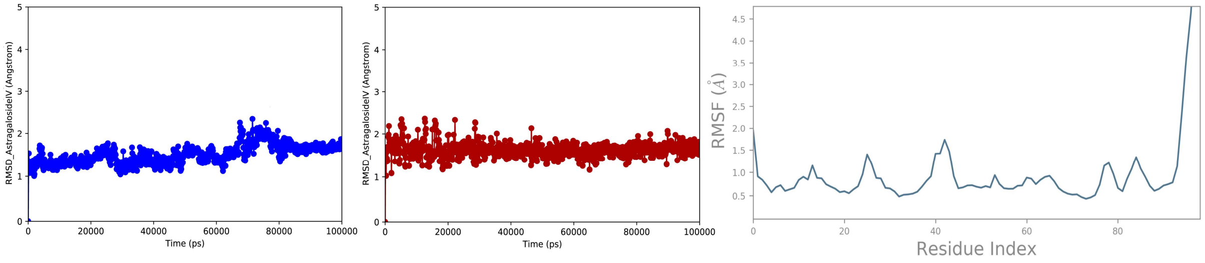

Continue

Irisin/Zoledronate

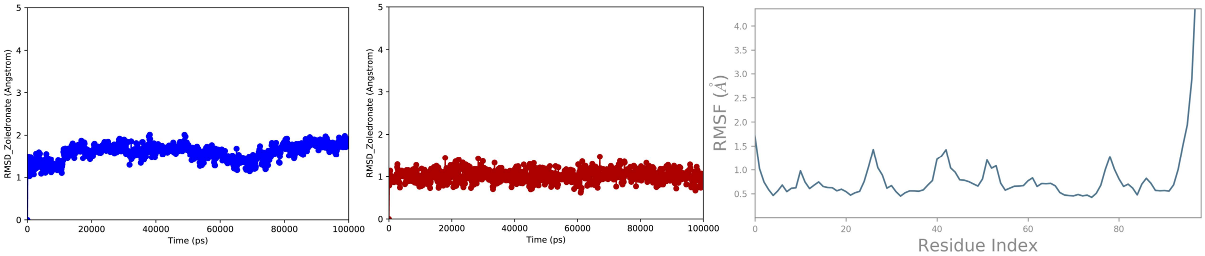

Irisin/Setmelanotide

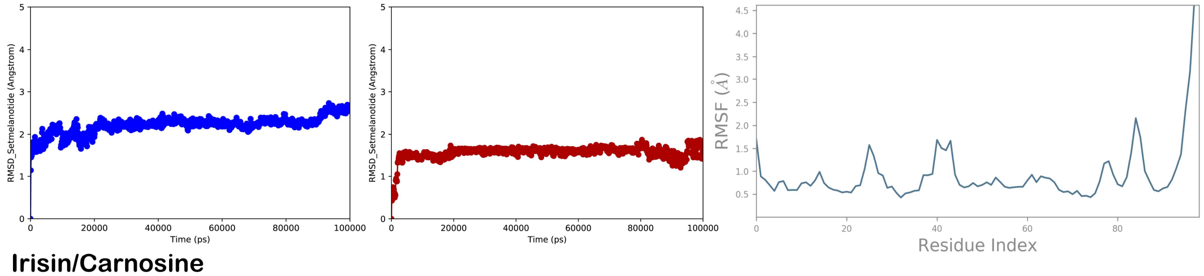

Irisin/Carnosine

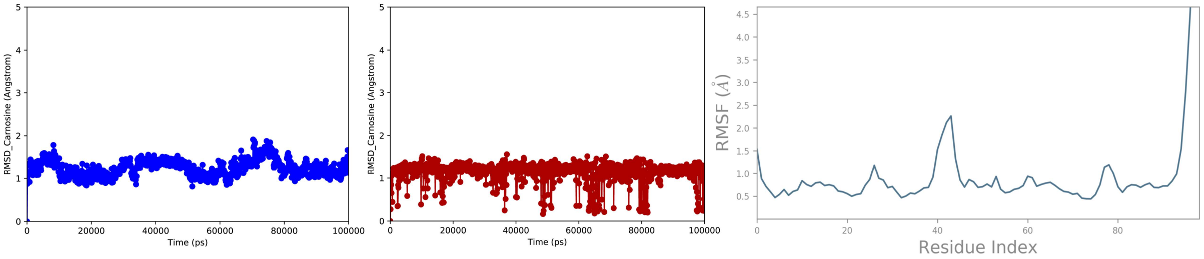

Irisin/Edotecarin

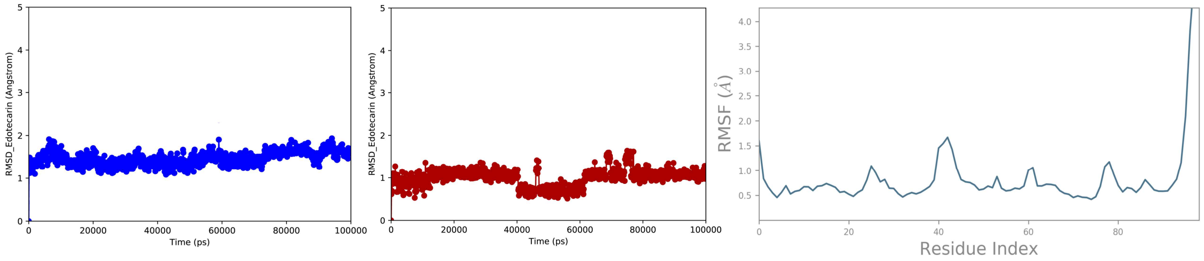

Irisin/Iopamidol

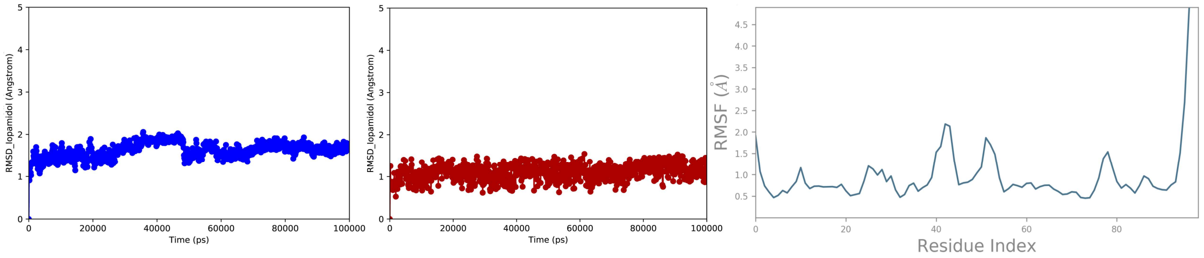

Irisin/Echinacoside

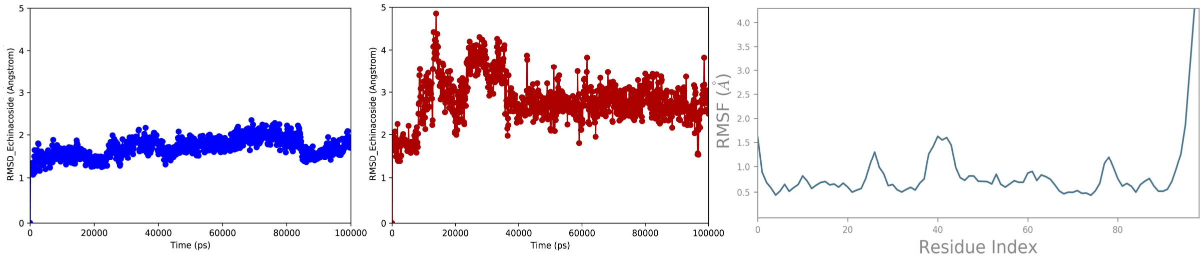

Continue

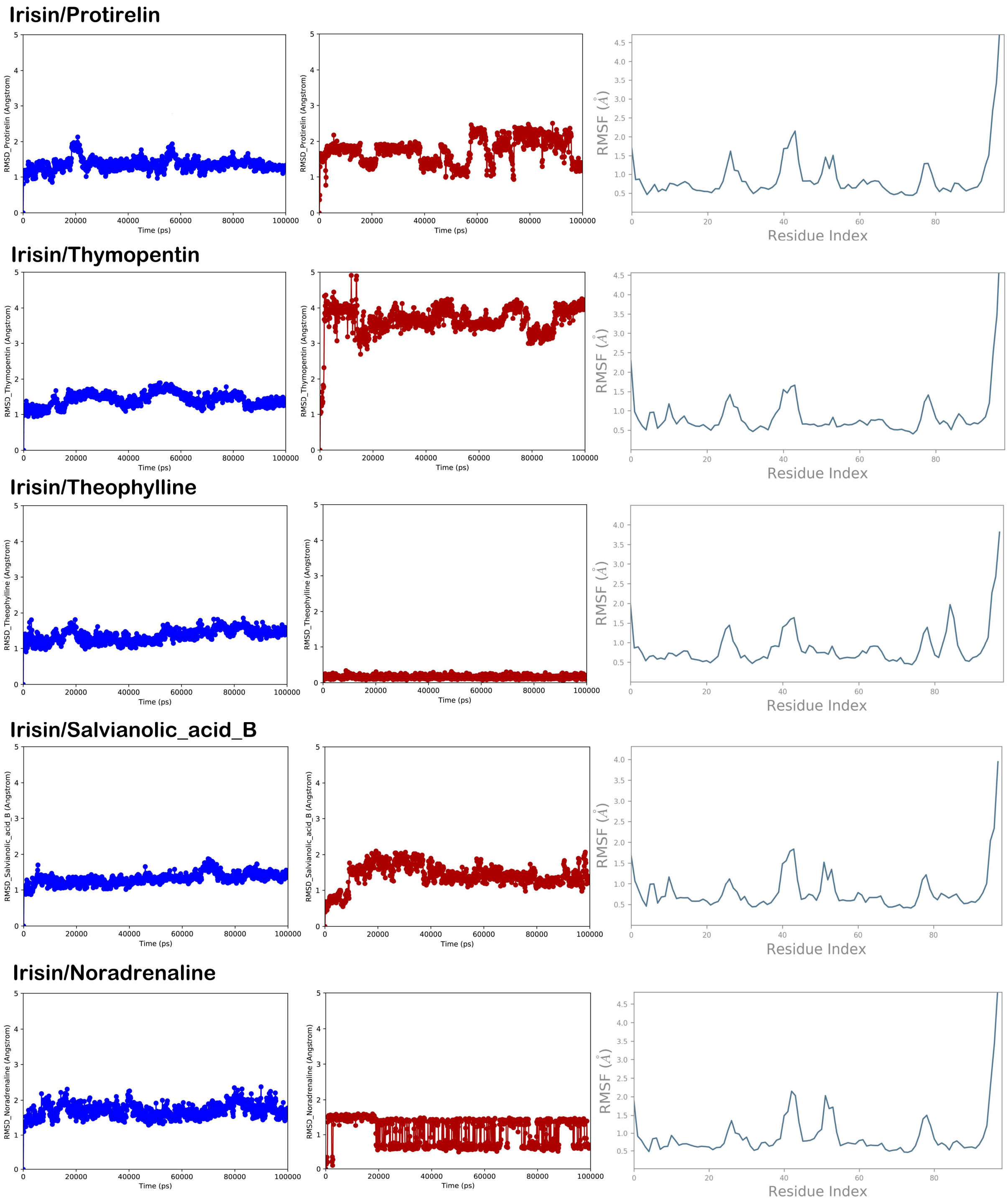

Figure S1. RMSD calculation (protein (blue line) and ligand (red line)) and RMSF evaluation for each complex, selected by docking studies, after 100 ns of MD simulation. Pictures were generated by Maestro (Maestro, Schrödinger LLC, release 2020-3).
